# A content analysis of women’s experiences of different models of maternity care: the Birth Experience Study (BESt)

**DOI:** 10.1186/s12884-023-06130-2

**Published:** 2023-12-15

**Authors:** Helen Pelak, Hannah G. Dahlen, Hazel Keedle

**Affiliations:** School of Nursing and Midwifery, Western Sydney University, Locked Bag 1797, 167 Great Western Highway, Blackheath, Penrith, NSW 2751 Australia

**Keywords:** Midwifery, Models of care, Continuity of care, Women’s experiences

## Abstract

**Background:**

Pregnancy, birth, and early parenthood are significant life experiences impacting women and their families. Growing evidence suggests models of maternity care impact clinical outcomes and birth experiences. The aim of this study was to explore the strengths and limitations of different maternity models of care accessed by women in Australia who had given birth in the past 5 years.

**Methods:**

The data analysed and presented in this paper is from the Australian Birth Experience Study (BESt), an online national survey of 133 questions that received 8,804 completed responses. There were 2,909 open-ended comments in response to the question on health care provider/s. The data was analysed using content analysis and descriptive statistics.

**Results:**

In models of fragmented care, including standard public hospital care (SC), high-risk care (HRC), and GP Shared care (GPS), women reported feelings of frustration in being unknown and unheard by their health care providers (HCP) that included themes of exhaustion in having to repeat personal history and the difficulty in navigating conflicting medical advice. Women in continuity of care (CoC) models, including Midwifery Group Practice (MGP), Private Obstetric (POB), and Privately Practising Midwifery (PPM), reported positive experiences of healing past birth trauma and care extending for multiple births. Compared across models of care in private and public settings, comments in HRC contained the lowest percentage of strengths (11.94%) and the highest percentage of limitations (88.06%) while comments in PPM revealed the highest percentage of strengths (95.93%) and the lowest percentage of limitations (4.07%).

**Conclusions:**

Women across models of care in public and private settings desire relational maternity care founded on their unique needs, wishes, and values. The strengths of continuity of care, specifically private midwifery, should be recognised and the limitations for women in high risk maternity care investigated and prioritised by policy makers and managers in health services.

**Trial registration:**

The study is part of a larger project that has been retrospectively registered with OSF Registries Registration DOI 10.17605/OSF.IO/4KQXP.

## Background

Pregnancy, birth, and early parenthood are significant life experiences impacting women and their families. In Australia, around 300,000 women give birth each year using public or private maternity service models [1]. While a ‘model of care’ is frequently used in healthcare to characterise the way health services are delivered, it is poorly understood and not easily defined [2, 3].

The most recent Australian data (2023) reported nearly 900 maternity models of care [1]. These models have been grouped into 11 major model categories based on three domains: the women a model is designed for; the carers working within the model; and how care is commonly provided. The most common model was *public hospital maternity care* which is fragmented in nature (40% of all models), meaning women can see a variety of health care providers at appointments and during labour and birth [1]. There are also a variety of high risk models (5% of models) that involve antenatal care provided to women with medical risk by public hospital health care providers (HCPs) that may include specialist obstetricians and/or maternal–fetal medicine subspecialists along with midwives [1]. Women may be able to access continuity of care (CoC) with a midwife through a public hospital in midwifery group practices (MGP) (15% of models) or through a privately practising midwife (PPM) (2% of models). Women may also access medical-led continuity of care with a private obstetrician (POB) (11% of models) and through shared care with a general practitioner (GPS) (14.6% of models) at a private or public hospital [1]*.*

A systemic review found women who received midwifery led continuity of care were less likely to experience intervention and more likely to report positive experiences of care than women who received care in other models [4]. In Australia, midwifery-led continuity of care has been recommended nationally due to its benefits in reducing adverse events and increasing positive experiences for women [4–7].

Women’s feedback, perspectives, and experiences of giving birth under different models of care provides crucial information and insights into how to improve the quality of maternity services [8]. The aim of this paper is to explore the strengths and limitations of Australian models of maternity care as voiced by women who completed an online survey and had a baby in Australia between 2016 and 2021.

## Methods

The data analysed and presented in this paper is from the Australian Birth Experience Study (BESt) which was a co-designed, online, national, cross-sectional survey undertaken between 9^th^ March and 30^th^ November 2021. The online survey consisted of 133 questions including demographic information, open and closed questions designed by the researchers and consumer reference group and the validated survey instruments Nijmegen Continuity Questionnaire [9], Mothers’ Autonomy in Decision Making (MADM) [10], Mothers on Respect index (MORi) [11] and The Mistreatment Index (MIST) [12]. Research team designed questions included open and closed questions previously used in national surveys that had utilised cognitive focus groups with the intended population, which demonstrated high content validity [13–15].

The survey was available in seven languages other than English: Arabic, Simplified Chinese, Hindi, Filipino, Persian, Thai and Vietnamese. The translations were undertaken by paid bilingual members of the cultural steering group. The validated survey instruments were not previously validated in the non-English languages and due to the low uptake of responses in the languages were unable to be validated. The survey was designed and distributed using Qualtrics software.

The BESt project was co-designed with a consumer reference group with representatives from ten Australian maternity and consumer advocacy organisations. The consumer reference group were involved in all aspects of the research process including survey development, piloting the survey and recruitment. Further information on survey development and co-design of the study can be found in Keedle et al. (2023) [16]. Ethical approval was obtained through the Western Sydney University Human Ethics Board, approval number: H14260.

### Participant recruitment

The online survey used non-probability, self-selection convenience sampling. The inclusion criteria were individuals who were able to read and write in English or the translated languages and had a baby in Australia between 2016 and 2021. The survey was open to any women who had a baby in the last 5 years in Australia, regardless of outcome, model of care or ‘risk’ status. There was a special pathway in the survey for women who had lost a baby so the questions would be less distressing. Women were asked to report on their most recent birth in the previous five years.

A social media page was formed that had recruitment posts in each language with the link to the survey landing page. Survey media campaigns in the form of social media advertising were utilised in each of the languages. The survey received over 12,000 partial responses resulting in 8,804 responses that were more than 75% completed, and 54,896 open text responses to 26 qualitative questions, from women in every State and Territory of Australia.

### Content analysis

A qualitative content analysis was undertaken on responses to one open-ended question requesting further information about their main health care provider/s: “*Do you have any further comments about your main health care provider/s?*” Incorporating qualitative questions into survey design provides insight into the experiences and reasoning behind decisions [17]. Content analysis uses systematic methods to categorise text and create coding frameworks to understand patterns and meaning from textual data and is particularly useful when dealing with large numbers of responses as occurred in this survey [18].

**Fig. 1:**
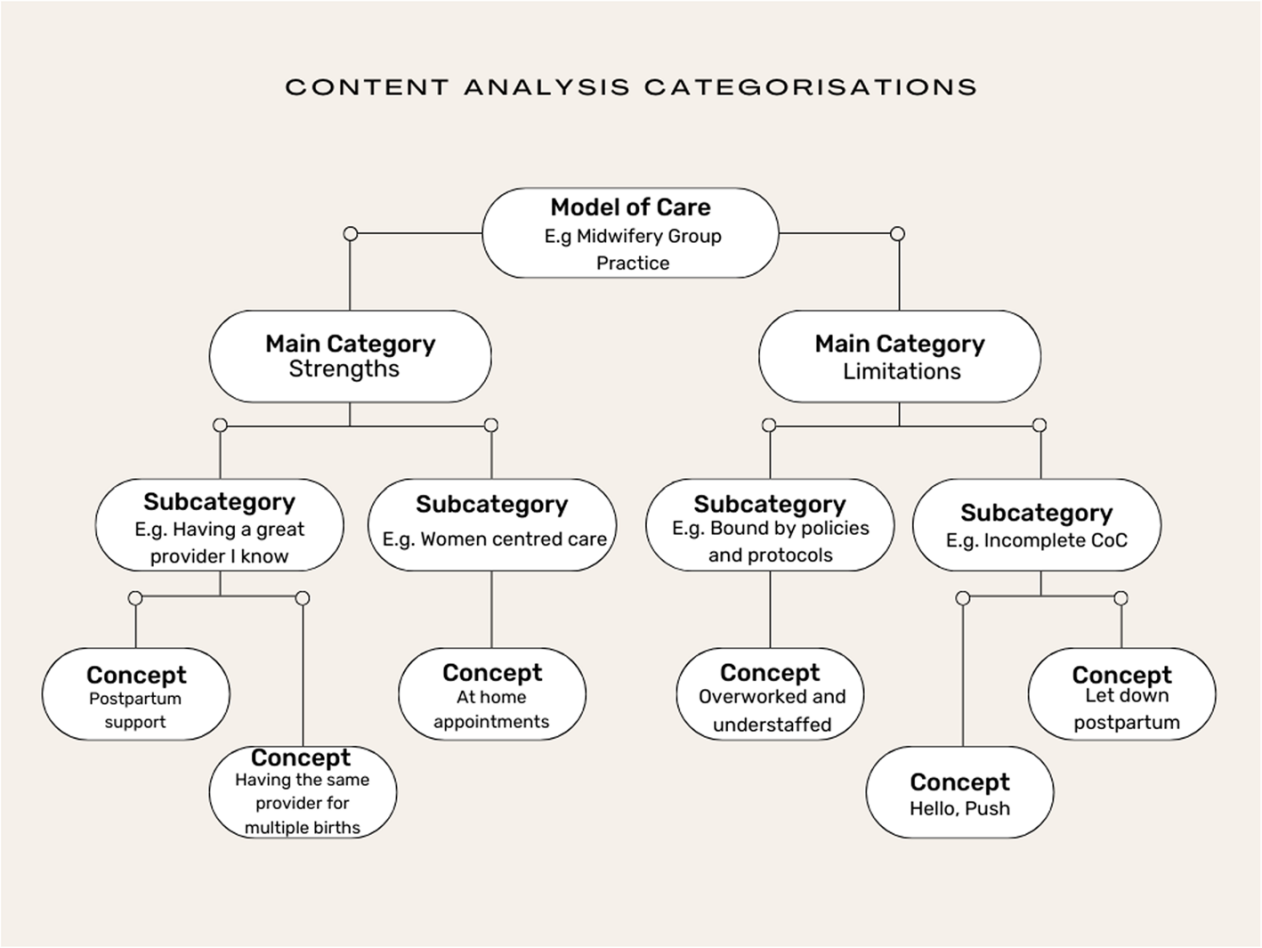
Content analysis categorisations

Firstly, the responses from the question were extracted from the survey dataset and separated into individual excel documents for the following models of care; public hospital care, high risk care, GP shared care, private obstetric care, midwifery group practice and privately practising midwifery care. The individual excel documents were uploaded to Nvivo. This was followed by an initial inductive content analysis with categories developed from the dataset [18, 19]. Items of coding were identified in each comment and some comments consisted of more than one item of coding. Following the first level of analysis of comments from each model of care a coding framework was developed as it was clear the main categories were grouped around the strengths and limitations of each model of care, as experienced by the participants. The coding framework consisted of three levels; main categories, subcategories and concepts, see Fig. 1. The frequency of the items of coding across the coding frame is presented as number of items of coding and percentage of frequency. This is also reported in previous content analysis papers [20–22].

Initial coding was undertaken by HP alongside weekly meetings with HK and HD to discuss and clarify categories. Final categorisation was agreed to by all authors. Participants were assigned identification numbers. The identification number is included in brackets following each item of coding.

### Reflexivity

Reflexivity is an important aspect of qualitative research to identify the positionality of the researchers and to ensure rigor in the research process [23, 24]. HP was a Fulbright scholar supervised by research mentors HK and HD. HP was provided with qualitative research training and attended regular meetings with the research team which provided opportunities for discussion and reflection. HK and HD are experienced midwifery researchers in qualitative methodologies. Having a non-midwife undertake the first level of analysis removed the assumptions the experienced midwives may have and led to robust and useful discussion, clarification and reflection.

## Results

### Participants

From 8,804 responses in the Birth Experience Study, there were 2,909 (33% of the BESt survey cohort) respondents who left comments in the following open-ended question requesting further information about their main health care provider/s: “*Do you have any further comments about your main health care provider/s?*”. There were 220 comments that provided no further information (e.g., No / N/A / Nope) and were removed from analysis which resulted in analysis of 2,689 comments. Four women commented in their own languages that differed from English (Persian (1), Thai (1), and Mandarin (2)) and were translated into English by members of the research cultural steering group. The content analysis resulted in 3,869 items of coding.

A maternity model of care is a recognised and standardised care pathway that identifies the health care providers, locations and structure of care delivered within that pathway [3, 25]. The models of care represented in the findings include standard public hospital care (SC), high-risk care (HRC), GP Shared care (GPS), Midwifery Group Practice (MGP), Private Obstetric (POB), and Privately Practising Midwifery (PPM) and the groupings are based on the Maternity Care Classification System [3].

Of women responding to the question on their main health care provider/s, 30.82% had standard maternity care and 9.52% attended high risk clinics in public hospitals; 8.81% had a GP shared care arrangement; 26.42% had continuity of care with a midwife through a public hospital; 8.95% received continuity of care with a privately practising midwife and 15.47% had continuity of care with a doctor (Table 1). Most women were 30-39yrs of age, were university educated and were born in Australia, further demographic information can be found in Table 1.

**Table 1:** Participant demographics

Demographics	Count (*n* = 2793)	(%) (*n* = 2793)
**Parity**		
Primipara	1308	46.83%
Multiparas	1485	53.17%
**Age range**		
Under 18–24	82	2.94%
25–29	532	19.05%
30–34	1185	42.43%
35–39	753	26.96%
40 +	241	8.63%
**Combined household income**		
Less than 40,000	70	2.51%
40,000–99,999	833	29.82%
More than 100,000	1759	62.98%
Prefer not to answer	131	4.69%
**Education**		
Year 12 or less	274	9.81%
Technical College (TAFE)* or diploma	549	19.66%
Undergraduate degree	1072	38.38%
Postgraduate qualification	898	32.15%
**Indigenous**		
No	2726	97.60%
Yes, Aboriginal	46	1.65%
Yes, Torres Strait Islander	2	0.07%
Yes, Both Aboriginal and Torres Strait Islander	2	0.07%
Prefer not to say	15	0.54%
Did not answer	2	0.07%
**Country of birth (Nationality)**		
Australian	2374	85.00%
European	191	6.84%
New Zealand	76	2.72%
North, Central and South American	53	1.90%
African and Middle Eastern	45	1.61%
North, South and Central Asian	53	1.90%
Melanesian, Papuan and Polynesian	1	0.04%
**Relationship status**		
Partnered	2662	95.31%
Unpartnered	123	4.40%
Other	8	0.29%
**Language other than English at home**		
No	2559	91.62%
Yes	234	8.38%
**Model of Care**		
Standard care (SC)	861	30.82%
High-Risk Care (HRC)	266	9.52%
Continuity of care with public midwife (MGP)	738	26.42%
Continuity of care with doctor (POB)	432	15.47%
GP shared care (GPS)	246	8.81%
Private midwife (PPM)	250	8.95%
No health care	0	0.00%
**Mode of birth**		
Vaginal birth	1716	61.44%
CS during labour	420	15.04%
Assisted Vaginal birth (forceps/vacuum)	353	12.64%
CS before labour	277	9.92%
Vaginal breech	27	0.97%
**Time since recent birth**		
Less than 6 months	670	23.99%
6 months – 1 year	502	19.97%
1 year – 2 years	705	25.24%
2 years to 3 years	459	16.43%
3 years to 4 years	239	8.56%
4 years to 5 years	208	7.45%
Did not Answer^a^	10	0.36%
**Birth Pre or During COVID-19**		
Pre COVID-19	1611	57.68%
During COVID-19	1172	41.96%
Did not Answer^a^	10	0.36%

^a^Time since birth question was not compulsory and could be skipped in the survey. The 10 identified didn’t answer the time since birth question but did leave a comment regarding their health care professional and were included in the analysis

### Qualitative findings-strengths and limitations relating to models of care

During the content analysis it was evident there were positive and negative items of coding related to each model of care and a coding framework was developed with the major categories of strengths and limitations. Each model of care differed in the numbers of items of coding about strengths and limitations (see Table 2). The PPM model had the highest percentage of items of coding about strengths (95.93%; *n* = 519) and lowest percentage of items of coding about limitations (4.07%; *n* = 22) and HRC had the lowest percentage of items of coding about strengths (11.94%; *n* = 56) and highest percentage of items of coding about limitations (88.06%; *n* = 413).

**Table 2:** Summary of strengths and limitations per model of care

Model of Care	Number of items of coding	Main Category	Number of items of coding	Percentage of total by model of care
Standard Care	844	Strengths	115	13.63%
		Limitations	729	86.37%
High Risk Care	469	Strengths	56	11.94%
		Limitations	413	88.06%
GP Shared	365	Strengths	52	14.25%
		Limitations	313	85.75%
Midwifery Group Practice	1063	Strengths	514	48.35%
		Limitations	549	51.65%
Private Obstetrician	587	Strengths	404	68.82%
		Limitations	183	31.18%
Privately Practising Midwife	541	Strengths	519	95.93%
		Limitations	22	4.07%

 The categories under strengths and limitations had similarities and differences across the models of care. For example, the subcategory ‘striking it lucky’ was found in three of the six models of care, and ‘bound by hospital policies and practices’ in all six models. There were also subcategories unique to a model of care and categorised under the strength or limitation major categories (Table 3 and 4).

**Table 3:** Distribution of strengths subcategories

Subcategories	Standard Care	High Risk Care	GP Shared	Midwifery Group Practice	Private Obstetrician	Privately Practising Midwife
Striking it lucky	✓	✓	✓			
Having a great provider I know			✓	✓	✓	✓
Access to a multi-disciplinary team		✓		✓	✓	
Woman-centered care				✓		✓
Individualised care around risk factors					✓	
Having the ‘gold standard’ of maternity care						✓

**Table 4:** Distribution of limitations subcategories

Subcategories	Standard Care	High Risk Care	GP Shared	Midwifery Group Practice	Private Obstetrician	Privately Practising Midwife
I felt completely anonymous	✓	✓	✓			
Lack of continuity of care was a major failing	✓	✓	✓			
Great divide between community and hospital			✓			
I wasn't able to form much of a meaningful relationship				✓		✓
Incomplete continuity of care				✓	✓	
Impersonal, clinical care					✓	
Bound by policies and practices	✓	✓	✓	✓	✓	✓

Each model of care will now be presented with a summary of the subcategories and concepts under the main categories of strengths and limitations. In each model of care the subcategories and concepts that are specific to that model will be discussed to prevent repetition. Subcategories and concepts introduced in previous models won’t be discussed in detail in subsequent models of care. A detailed list of all subcategories and concepts are included in Table 5 - Complete content analysis framework.

**Table 5:** Complete content analysis framework

Model of Care	Number of quotes	Frequency of total	Main Category	Number of quotes	Frequency of total	Subcategory	Number of quotes	Frequency of total	Concept	Number of quotes	Frequency of total
**Standard Care**	**844**	**21.81%**	**Strengths**	**115**	**2.97%**	**Striking it Lucky**	**115**	**2.97%**	Everyone was great	58	**1.50%**
									Having a great provider I know	27	**0.70%**
									Having a student midwife	19	**0.49%**
									Working in the birthing world	11	**0.28%**
			**Limitations**	**729**	**18.84%**	**Lack of continuity of care was a major failing**	**488**	**12.61%**	I would have loved continuity of care	350	**9.05%**
									Hello, Push	57	**1.47%**
									I had to keep repeating my story	50	**1.29%**
									Offered contradictory medical advice	28	**0.72%**
									Letdown postpartum	3	**0.08%**
						**I felt completely anonymous**	**175**	**4.52%**	My concerns were dismissed	71	**1.84%**
									I did not feel heard	25	**0.65%**
									They were constantly trying to find something wrong	21	**0.54%**
									Gaps in care	21	**0.54%**
									I would never go public again	16	**0.41%**
									Provider was disrepectful, coercive	7	**0.18%**
									Fight for VBAC	6	**0.16%**
									Cascade of interventions	5	**0.13%**
									Ongoing issues processing and healing from the birth	3	**0.08%**
						**Bound by policies and protocols**	**66**	**1.71%**	COVID-19 influence	32	**0.83%**
									Overworked and understaffed	13	**0.34%**
									I had a bit of a different method of care due to living remotely	12	**0.31%**
									Excessive waiting times	9	**0.23%**
**High Risk Care**	**469**	**12.12%**	**Strengths**	**56**	**1.45%**	**Striking it Lucky**	**50**	**1.29%**	Everyone was great	22	**0.57%**
									Having a great provider I know	21	**0.54%**
									Working in the birthing world	5	**0.13%**
									Having a student midwife	2	**0.05%**
						**Access to a multi-disciplinary team**	**6**	**0.16%**			
			**Limitations**	**413**	**10.67%**	**Lack of continuity of care was a major failing**	**245**	**6.33%**	I would have loved continuity of care	112	**2.89%**
									Dangers of fragmented care	80	**2.07%**
									I had to keep repeating my story	31	**0.80%**
									Hello, Push	12	**0.31%**
									Letdown postpartum	10	**0.26%**
						**I felt completely anonymous**	**107**	**2.77%**	I've never felt so disregarded in my own healthcare	77	**1.99%**
									My concerns were dismissed	19	**0.49%**
									Cascade of interventions	8	**0.21%**
									Fight for VBAC	3	**0.08%**
						**Bound by policies and protocols**	**61**	**1.58%**	I was denied continuity of care	28	**0.72%**
									They unnecessarily deemed me high risk	15	**0.39%**
									COVID-19 influence	8	**0.21%**
									My provider prior to transfer was still the person I turned to	8	**0.21%**
									Excessive waiting times	2	**0.05%**
**GP Shared**	**365**	**9.43%**	**Strengths**	**52**	**1.34%**	**Striking it Lucky**	**23**	**0.59%**	Everyone was great	17	**0.44%**
									Working in the birthing world	4	**0.10%**
									Having a student midwife	2	**0.05%**
						**Having a great provider I know**	**29**	**0.75%**	Having a great GP I knew and trusted prior to pregnancy	27	**0.70%**
									Postpartum support	2	**0.05%**
			**Limitations**	**313**	**8.09%**	**I felt completely anonymous**	**93**	**2.40%**	I was just a number	69	**1.78%**
									I got lost in the system	11	**0.28%**
									Offered contradictory medical advice	9	**0.23%**
									Cascade of interventions	4	**0.10%**
						**Great divide between community and hospital**	**130**	**3.36%**	Two different experiences	60	**1.55%**
									Communication breakdown	43	**1.11%**
									My GP didn't know a whole lot about maternity care	21	**0.54%**
									I didn't know who I could go to when I needed to ask questions	6	**0.16%**
						**Lack of continuity of care was a major failing**	**60**	**1.55%**	I would have loved continuity of care	28	**0.72%**
									Hello, Push	18	**0.47%**
									I had to keep repeating my story	6	**0.16%**
									Letdown postpartum	8	**0.21%**
						**Bound by policies and protocols**	**30**	**0.78%**	COVID-19 influence	19	**0.49%**
									Overworked and understaffed	11	**0.28%**
**Midwifery Group Practice**	**1063**	**27.47%**	**Strengths**	**514**	**13.29%**	**Access to a multi-disciplinary team**	**38**	**0.98%**	Everyone was great	16	**0.41%**
									Working in the birthing world	9	**0.23%**
									Having access to MGP care	7	**0.18%**
									Having a student midwife	6	**0.16%**
						**Having a great provider I know**	**325**	**8.40%**	Having my own midwife	225	**5.82%**
									Having the same provider for multiple births	49	**1.27%**
									Postpartum support	16	**0.41%**
									Having an inclusive relationship with my provider	15	**0.39%**
									Far better experience than last time	12	**0.31%**
									My midwife was amazing, especially during covid restrictions	8	**0.21%**
						**Woman-centered care**	**151**	**3.90%**	Respectful care	63	**1.63%**
									Skilled and knowledgeable care	34	**0.88%**
									This model should be the standard and accessible to all	26	**0.67%**
									Bolstering confidence in women's birthing abilities	14	**0.36%**
									At-home appointments	14	**0.36%**
			**Limitations**	**549**	**14.19%**	**I wasn't able to form much of a meaningful relationship**	**241**	**6.23%**	Felt more disjointed than I had expected	139	**3.59%**
									I didn't really 'click' with my midwife	59	**1.52%**
									Minimal emphasis on birth preferences	23	**0.59%**
									I had to keep repeating my story	10	**0.26%**
									Offered contradictory medical advice	10	**0.26%**
						**Bound by policies and protocols**	**149**	**3.85%**	I did not get to choose my MGP care provider	40	**1.03%**
									Overworked and understaffed	38	**0.98%**
									Restrictive hospital policies	32	**0.83%**
									COVID influence	20	**0.52%**
									Accepted into MGP later in pregnancy	19	**0.49%**
						**Incomplete continuity of care**	**159**	**4.11%**	Hello, Push	82	**2.12%**
									I would have loved continuity of care	64	**1.65%**
									Letdown postpartum	13	**0.34%**
**Private Obstetrician**	**587**	**15.17%**	**Strengths**	**404**	**10.44%**	**Access to a multi-disciplinary team**	**57**	**1.47%**	Access to mdwifery care	42	**1.09%**
									Working in the birthing world	7	**0.18%**
									I was able to contact the maternity ward 24 h a day	6	**0.16%**
									Having a student midwife	2	**0.05%**
						**Having a great provider I know**	**213**	**5.51%**	Having a doctor I know	133	**3.44%**
									I felt they understood my priorities, concerns	35	**0.90%**
									Respectful care	17	**0.15%**
									Far better experience than last time	9	**0.23%**
									Having an inclusive relationship with my provider	7	**0.18%**
									Having the same provider for multiple births	6	**0.16%**
									I felt safe	6	**0.16%**
						**Individualised care around risk factors**	**134**	**3.46%**	Skilled and knowledgeable care	92	**2.38%**
									Around the clock care	24	**0.62%**
									Healing from a previously traumatic birth experience	8	**0.21%**
									Support for VBAC	6	**0.16%**
									Option for extra monitoring and scans	4	**0.10%**
			**Limitations**	**183**	**4.73%**	**Impersonal, clinical care**	**111**	**2.87%**	My OB didn't know who I was	30	**0.78%**
									My concerns were dismissed	29	**0.75%**
									I didn't feel like anything was explained well	13	**0.34%**
									I ended up with the birth that was convenient for my Obstetrician	13	**0.34%**
									Cascade of interventions	8	**0.21%**
									I felt like I was part of a system	8	**0.21%**
									Fight for VBAC	6	**0.16%**
									Each appointment and scan was very short	4	**0.10%**
						**Incomplete continuity of care**	**52**	**1.34%**	Hello, Push	37	**0.96%**
									Letdown postpartum	15	**0.39%**
						**Bound by policies and protocols**	**20**	**0.52%**	Overworked and understaffed	9	**0.23%**
									COVID influence	7	**0.18%**
									Expensive, even with private cover	4	**0.10%**
**Privately Practicing Midwife**	**541**	**13.98%**	**Strengths**	**519**	**13.41%**	**Having a great provider I know**	**246**	**6.36%**	Having my own midwife	143	**3.70%**
									Far better experience than last time	24	**0.62%**
									I felt supported throughout my pregnancy, birth, and my postpartum period	22	**0.57%**
									Having an inclusive relationship with my provider	18	**0.47%**
									Having the same provider for multiple births	13	**0.34%**
									She is a part of my family now	9	**0.23%**
									Healing from a previously traumatic birth experience	9	**0.23%**
									My midwife was amazing, especially during covid restrictions	8	**0.21%**
						**Having the gold standard of maternity care**	**163**	**4.21%**	I couldn’t rate my midwife more highly	129	**3.33%**
									Best value for money every spent	15	**0.36%**
									This model should be the standard and accessible to all	10	**0.26%**
									Postpartum support	9	**0.23%**
						**Woman-centered care**	**110**	**2.84%**	Respectful care	52	**1.34%**
									I felt safe	18	**0.47%**
									Skilled and knowledgab le care	13	**0.34%**
									Bolstering confidence in women's birthing abilities	12	**0.31%**
									At-home appointments	10	**0.26%**
									Around the clock care	5	**0.13%**
			**Limitations**	**22**	**0.57%**	**I wasn't able to form much of a meaningful relationship**	**18**	**0.47%**	I had big hopes for my private midwife	7	**0.18%**
									Impersonal care concerning birth preferences	6	**0.16%**
									Hello, Push	5	**0.13%**
						**Bound by policies and protocols**	**4**	**0.10%**	COVID influence	2	**0.05%**
									They weren't allowed at the birth	2	**0.05%**

### Standard care

Women within the standard model of care highlighted experiences of fragmented and impersonal care. Standard care had one subcategory in the strengths category and three subcategories in the limitations category.

#### Strengths

In the subcategory ‘Striking it lucky’, women described being fortunate enough to form a relationship with their provider despite being in a fragmented model. The concept *‘Having a great provider I know’* highlighted how some women found midwives that would go beyond their work requirements to provide as much continuity as possible*, “Towards the latter part of pregnancy one of the clinical midwives arranged for me to see her for the remainder of my appointments and also visited me post birth while I was recovering in hospital (completely above & beyond her actual job requirements but so very appreciated).”* (ID 2467).

Some women were ‘*Working in the birthing world’* such as midwives or doctors, and received standard care, but they were able to negotiate a modified continuity of care through friends and colleagues*, “As I am a midwife myself, I was able to have a trusted colleague and friend provide antenatal care even though I technically did not qualify for midwifery care due to increased BMI.”* (ID 1746).

Some women found that despite being in SC, they received continuous care from ‘*having a student midwife*’.

*“Whilst I did not have a "main health care provider", I was lucky enough to meet a 3rd year student midwife who from about 30 weeks joined me on the remainder of my journey. This was the only consistent care I received and without a doubt the best part of my journey.”* (ID 2502).

In the concept ‘*Everyone was great’*, positive interactions and feelings of being well looked after, despite receiving fragmented care and a limited ability to form relationships with providers, were highlighted. *“Even though I didn’t have one midwife the whole way through all the midwives at my hospital were very knowledgeable and understanding.”* (ID 1837).

#### Limitations

The largest subcategory expressing limitations in the SC group was ‘Lack of continuity of care was a major failing’. This subcategory consisted of five concepts related to the impacts of not receiving continuity of care. In the concept ‘*I would have loved continuity of care’* women expressed their wish for continuity of care, including in the postnatal period in the concept *‘Letdown postpartum’*. Some women were unaware of continuity models of care or unable to access the model. *“I so badly wanted to have continuity of care but wasn't able to access the program due to oversubscription.”* (ID 2086). Other women described not being told they had this option.

*“I originally would have liked continuity of care but was not even given it as an option at booking-in, nor was I informed of that option by GP. I did not know that “midwifery group practice” existed or what it was until I heard about it much later in pregnancy and had wondered how to get into it, because those care options were not mentioned to me at booking-in.”* (ID 2025).

In the concept ‘*I had to keep repeating my story’*, women expressed feelings of frustration and exhaustion in having to repeat their medical history. *“I dreaded going to the hospital for appointments because I knew I would have to go over my traumatic previous birth with whoever I was with that day.”* (ID 2824). Women also noted that a lack of continuity could result in being ‘*Offered contradictory medical advice’* which could often be conflicting to other providers advice*.*

*“Different doctor every visit. Different information was given for VBAC. Very hard to*

*navigate. Cannot imagine if there was a language barrier or vulnerable.”* (ID 1804).

The lack of continuity during labour and birth led to the concept ‘*Hello, Push’*, and focused on their disappointment in not knowing their providers during labour. *“My midwife who did my antenatal classes, was not there for my birth, which was extremely disappointing.”* (ID 2432).

In the subcategory ‘I felt completely anonymous’ women described feeling like part of an automated system with minimal personal connection and limited respect for their wishes and dignity. This subcategory included nine concepts that ranged from ‘*My concerns were dismissed*’ to ‘*Cascade of intervention’*. One woman stated that she *“felt very much on the conveyor belt from day 1.”* (ID 1942). Another woman detailed her birth experience as feeling though she was on a ‘production line’*,*

*“I saw someone different at nearly every appt. They were usually lovely, but there was no consistency and I often felt like I was on a production line.”* (ID 2075).

Repeatedly, women described feeling as if they were on a production line with one woman commenting that *“all I felt like was a number, not a human.”* (ID 2116).

In the subcategory ‘Bound by policies and protocols’ women discussed factors that limited care that were outside the control of providers. Concepts included *‘Overworked and understaffed’“Our hospital’s midwifery department is so understaffed that the level of care is not adequate.”* (ID 2355), *‘Excessive waiting times’*, *‘I had a different method of care due to living remotely’* and *‘COVID-19 influence’*. One woman stated, *“Due to Covid restrictions some appointments were over the phone and the later ones were limited to ten minutes.”* (ID 1835).

### High-risk

Women in high-risk care (HRC) models commented on the dangerous implications of fragmented, impersonal care that included missed health complications, having to repeat past and sometimes traumatic birth experiences in every appointment and being offered contradictory medical advice. The high-risk model had the lowest percentage of items of coding mentioning strengths in care (11.94%) and the highest percentage of items of coding expressing limitations in care (88.06%).

#### Strengths

The subcategories and concepts identified in this model that were also present in standard care was the subcategory ‘Striking it lucky’ and it’s concepts *‘Everyone was great’*, *‘Having a great provider I know’*, *‘Having a student midwife’* and *‘Working in the birthing world’.* A subcategory identified that was specific to the high risk model was ‘*Access to a multi-disciplinary team’* where a few women noted they had access to a variety of providers due to their risk factors and this included an ease in the transfer of information during shared appointments.

*“Due to my complications, I had a main OB, haematologist, and was with the high-risk midwives’ team. I had very regular appointments and scans. My OB and haematologist ran shared appointments multiple times throughout my pregnancy to discuss haematology side of things.”* (ID 2547).

#### Limitations

High risk care consisted of the same three subcategories that were also present in standard care; ‘Lack of continuity of care was a major failing of this pregnancy’, ‘I felt completely anonymous’ and ‘Bound by policies and protocols’.

‘Lack of continuity of care was a major failing of this pregnancy’ consisted of five concepts which highlighted a strong desire for personal connection in care. The specific concept that related to high risk care was* ‘Dangers of fragmented care’.*

The lack of continuity of care was strongly noted in the concept *‘Dangers of fragmented care***’** in which some women noted feelings of fear and lack of safety in receiving conflicting medical advice.

*“… from 25 weeks I was having appointments weekly. During that time, I had multiple doctors all with differing opinions which was not only stressful for me but caused issues for me towards the end of my pregnancy that could have seen me lose my baby.”* (ID 2778).

In the subcategory ‘I felt completely anonymous’ there was one concept specific to high risk care; ‘*I never felt so disregarded in my own healthcare’.* Here women detailed experiences of impersonal care and feelings that their concerns were dismissed. One woman commented, *“I was just a number to them—I don’t think they even knew my name. They saw ‘previous caesarean’ on my file and that was enough for them to bully me through the entire process.”* (ID 2660). Another woman stated, *“The experience was very impersonal, their focus was my cervix, not preparing me for birth.”* (ID 185).

In the subcategory ‘Bound by policies and protocols’ the concepts that were unique to high risk care were *‘I was denied continuity of care’, ‘They unnecessarily deemed me high risk’* and *‘My provider prior to transfer was still the person I turned to’.* Women voiced feelings of frustration in becoming ineligible for continuity of care programs once their pregnancy was determined ‘high-risk in the concept *‘I was denied continuity of care’*.

*“I was told I wasn’t eligible for the midwife program as I had gestational diabetes and therefore higher risk. However, that meant I saw a different OB every appointment which I see as disappointing if I was higher risk.”* (ID 2161).

One woman noted, *“My GP was fantastic, unfortunately she had to pass me onto hospital care once I developed preeclampsia.”* (ID 226)**.** Women were also transferred from MGP care.

*“Continuity of care with a primary midwife was amazing, however when the pregnancy became high risk and transferred to the in hospital, I saw someone different each time and the experience was not nearly as personal or reassuring.”* (ID 394).

In the concept *‘they unnecessarily deemed me high risk’* women felt that the reasons they were allocated the high risk model of care were not clear or sufficient enough to be in the clinic, which caused extra frustration and confusion; *“I was repeatedly told by the obs that I wasn't technically high risk however they denied my repeated requests to continue my care under a midwifery model”* (ID144).

*“Whilst I was technically high risk due to two previous inductions due to high blood pressure I was on the low end of high risk. I was too risky for the regular midwife clinic but not interesting enough to require in depth long appointments.”.* (ID 283).

However, some women were able to maintain communication with their provider who cared for them prior to being transferred into high-risk care in the concept *‘My provider prior to transfer was still the person I turned to’, “My midwife was still called in to induce me and came back the next morning for my labour. I was so scared and feeling so unwell so seeing her face for the induction and then labour was so amazing.”* (ID 568). Some women found continuity of care by hiring private care. *“I hired a private midwife at 32 weeks so I could have some continuity in care as so many things were missed in my care through the public hospital (high risk with no midwife care).”* (ID 2834).

### GP shared care

Women who received GP Shared Care model (GPS) care described two contrasting aspects: continuity of care (CoC) with their GP in their community, and confusion and feeling lost within the hospital where they never saw a consistent provider.

#### Strengths

GP shared care consisted of two subcategories, ‘Striking it lucky’ and 'Having a great provider I know’. There were no additional concepts for this model of care in the subcategory ‘Striking it lucky’*,* however the subcategory 'Having a great provider I know’ consisted of two concepts specific to GP shared care.

In the subcategory 'Having a great provider I know’ women identified the benefit of *‘Having a great GP I knew and trusted prior to pregnancy’* which reflected the benefits of women’s relationships with their GPs that began prior to becoming pregnant. One woman noted,

*“My 'main provider' was my GP. He has been my GP my entire life, therefore knew me well and respected my decisions.”* (ID 2859).

Women under GPS care also discussed the benefits of in-community relationships when navigating fragmented hospital care.

*“… the hospital staff I interacted with were very different - midwives/doctors, etc - always different, hard to get follow up, often had to personally call to request things sent to my GP when they knew I was doing shared care. The hospital was difficult to work with but my GP was great at helping me navigate the system.”* (ID 112).

A couple of women identified the benefit of continuing to see their GP following their pregnancy in the concept ‘Postpartum support'. Some women who did not know their GP prior to pregnancy decided to continue care post-birth, *“I had to have a new GP partway through this last pregnancy, but I have liked her so much that I continue to see her now.”* (ID 142).

#### Limitations

There were four subcategories in GP shared care limitations. The subcategory specific to GP shared care was ‘Great divide between community and hospital’.

In the subcategory ‘Great divide between community and hospital’, which consisted of four concepts, women noted the care they received with their GP differed significantly from the care they received in the hospital in the concept ‘*Two different experiences’*.

*“My GP was my main care provider. She is wonderful however not involved in my birth or birthing education. My experience with the hospital was very different.”* (ID 166).

*‘Communication breakdown’*, where they had to repeat conversations they had with their GP in their hospital appointments due to the limited transfer of information,

*“I constantly had to repeat my circumstances and thought this info would have been passed on. It was frustrating and made me feel unimportant.”* (ID 115).

Some women also mentioned feeling their GP did not have adequate knowledge of pregnancy to provide relevant information and support in the concept *‘My GP didn’t know a whole lot about maternity care’*.

*“I didn't feel like my GP was equipped to deal with some of the complications I had during my pregnancy. I found it difficult to get the right information/support. It was a pregnancy after miscarriage, and I had significant bleeding/cramping which was a very stressful time.”* (ID 123).

A few women found the model of care confusing in the concept *‘I didn’t know who I could go to when I needed to ask questions’.*

*“I didn't know who I could go to when I needed to ask questions. When I needed a certificate to be able to fly for work the Dr was very condescending and rude.”* (ID 56).

### Midwifery group practice care

Women who received Midwifery Group Practice (MGP) care highlighted experiences of individualised care that centred their preferences and autonomy and the relationship they formed with their midwives that continued to develop after they gave birth. However, women noted this care was significantly impacted by hospital policies and protocols, and repeatedly, women mentioned feeling letdown in their expectations for continuity of care.

#### Strengths

Midwifery Group Practice had four strength categories that were present in previous models of care, ‘Access to a multi-disciplinary team’ and ‘Having a great provider I know’. There was one subcategory distinct to the continuity of care models, including MGP, which was ‘Woman-centered care’.

There were five concepts in the subcategory ‘Woman-centered care’. In the concept *‘Respectful care’* women reported that their midwife valued their preferences and autonomy, *“She was extremely supportive of my vbac [vaginal birth after caesarean section] wishes and was a huge advocate and encourager of mine throughout my entire pregnancy.”* (ID 922)*.* While another said, *“Empowered me [midwife] to make my own decisions throughout my pregnancy. She explained the consequences and my options, but it was up to me what to do”* (ID 415).

Some women found their midwives provided ‘*Skilled and knowledgeable care*’ which resulted in feelings of being well cared for and safety.

*“My midwife was experienced, knowledgeable, supportive, informative, timely, and contactable. Our developed rapport meant that I trusted her opinion, advice, and recommendations. This allowed me to make informed choices regarding my care and to feel safe and secure during my pregnancy, labour, and post-partum period.”* (ID 831).

In the concept *‘This model should be the standard and accessible to all’* women shared their thoughts on how MGP should be available for all who are accessing maternity care.

*“All women should have the opportunity of a Continuity program, helps reduce medical interventions by a lot, also helps that you have a midwife that knows you from first step to last.”* (ID 8).

In the concept, *‘Bolstering confidence in women’s birthing abilities’* women noted feeling empowered by their midwife to trust themselves and their abilities, “*She trusted me, and my birthing capabilities and I completely trusted her.”* (ID 911)*.* Another said,

*“I cannot speak highly enough of Midwifery Group Practice. I felt safe, loved, advocated for, and protected. They helped me transition into motherhood with confidence and trust in myself. I wouldn’t hesitate to use the same model of care next time!”* (ID 474).

Some women detailed their experiences of having *‘At-home appointments’* as a significant factor in feeling safe, comfortable, and well cared for. One woman commented, *“I loved that they came to my home for appointments, took the time to properly listen to me and knew me and my anxieties really well.”* (ID 719)*.* In the following comment, the woman describes the environment of at-home appointments and the ability to form a deeper relationship with their midwife in the process.

*“I would prepare coffee or tea and snacks, and my husband and son would all sit around the table chatting with our midwives for 1-2 hours. This made for a comfortable visit where they felt like an extension of our family at times, and we were all on this beautiful journey together.”* (ID 875).

#### Limitations

Midwifery group practice had one subcategory in the limitations category that was similar to previously reported models of care, ‘Bound by hospital policies and procedures’ and two subcategories that were found in the continuity models *‘Incomplete continuity of care’ and ‘I wasn’t able to form much of a meaningful relationship with anyone’.*

In the subcategory expressing limitations in the MGP group, *‘I wasn’t able to form much of a meaningful relationship with anyone’, there were two concepts that have been discussed in standard care, ‘I had to keep repeating my story’ and ‘Offered contradictory medical advice’ and three concepts found in continuity models ‘Felt more disjointed than I had expected’, ‘I didn’t really click with my midwife’ and ‘Minimal emphasis on birth preferences’.*

In the concept ‘*Felt more disjointed than I had expected’* women noted feelings of disappointment for not having received the continuity of care they had expected.

*“I was enrolled in a continuity of care program with a birth centre and was meant to have the same midwife throughout. However, due to staffing changes, I was assigned 3 different midwives throughout my pregnancy and my actual birth was attended by a different 4th midwife as the assigned midwife was on leave. It was a disappointing outcome for a continuity of care program.”* (ID 2846).

In the concept *‘I didn’t really click with my midwife’ *women identified a lack of depth and connection to the continuity relationship which impacted their overall experience.

*“I was in a continuity care program, so having the same midwife was great. However, I didn’t really “click” with my midwife, and would have liked to have been able to choose a midwife with whom I felt more connection.”* (ID 278).

*“Despite ‘best practice’ care I often felt my questions weren’t answered and didn’t have a good connection to my main care provider. Everything was done by the book in terms of follow up and care however the overall impression was of something missing.”* (ID 68).

Some women in MGP felt unprepared for their birth in the concept *‘Minimal emphasis on birth preferences’.*

*“Group education was provided at each appointment. Some of the resources were a bit outdated. There was not a lot of emphasis/discussion surrounding birth preferences.” (*ID114).

### Private obstetrician care

Women in Private Obstetric (POB) care highlighted their relationships with their doctors who were knowledgeable and attuned to potential health complications and past medical history. Items of coding also expressed women’s experiences of impersonal, medical care that centred their doctor’s opinions in care decisions.

#### Strengths

There were three subcategories in the strengths of Private Obstetric model of care, two that have been previously discussed *‘Access to a multi-disciplinary team’ and ‘Having a great provider I know’. There was one subcategory that was unique to POB, ‘Individualised care around risk factors’* which had five concepts.

In the concept *‘Skilled and knowledgeable care’* women felt they were in capable hands with their POB who provided around-the-clock care and reassurance.

*“I felt very confident in his decisions because I trusted that he was an expert. He did things by the book which may not have always been tailored to me personally. But I was okay with that.”* (ID 1182).

In the concept *‘Around the clock care’* women identified the benefit of being able to contact their doctor of private hospital maternity ward at any time to answer questions or give reassurance.

*“After several second trimester miscarriages I was very anxious during this pregnancy, my Dr provided his mobile number and I could call anytime I was worried. On more than one occasion I called as I was worried I hadn't felt baby move and he always had me come in straight away for an ultrasound to check the heart beat, not matter day or time. He had cared for me for all my losses and knew my history so was very compassionate and understanding.”* (ID 4).

Some women mentioned the availability for extra monitoring was the defining benefit in their care in the concept *‘Option for extra monitoring and scans’.*

*“After several second trimester miscarriages I was very anxious during this pregnancy, my dr provided his mobile number and I could call anytime I was worried. On more than one occasion I called as I was worried, I hadn't felt baby move and he always had me come in straight away for an ultrasound to check the heartbeat, no matter day or time.”* (ID 1077).

In the concept *‘Healing from a previously traumatic birth experience’* women identified the benefit of having a POB for their next birth, *“She was really great at looking after my mental health as well because she knew I had birth trauma from my previous birth.”* (ID 289).

*“Following a very eventful first birth by emergency c-section under GA with an OB who was ok but not brilliant, I was able to find a new OB for my second pregnancy who was able to support me in all the ways that were important for me to feel empowered for my second birth.”* (ID 46).

Some women identified how their POB supported their birth after caesarean choices in the concept *‘Support for VBAC’ I felt supported and trusted my private OB, who had performed my first C section by my choice, and then provided my care when I had a VBAC (but was unfortunately not at the birth).” (ID 98).*

#### Limitations

There were three subcategories in the limitations category for POB, two that were discussed in previous models ‘Incomplete continuity of care’ and ‘Bound by policies and procedures’ and one unique to POB ‘Impersonal, clinical care’. The subcategory *‘Impersonal, clinical care’* consisted of eight subcategories, three concepts were present in previous models of care and five concepts were specific to private obstetric care.

In the concept ‘*My OB didn't know who I was’* women highlighted the challenges of a lack of relational care. *“It felt like a checklist that he had to go through each visit rather than getting to know me or how I was.”* (ID 163).

Some women found a lack of information and options in the concept *‘I didn't feel like anything was explained well’*, *“I didn't feel like anything was explained well… a lot of the time I was told things rather than given the options or information.”* (ID 365).

Other women found POB care resulted in a lack of options related to birth in the concept *‘I ended up with the birth that was convenient for my Obstetrician*’.

“*My Obstetrician was very conventional in regards to his practice. He always provided an opportunity for me to ask questions in regards to my care and birthing options, however I failed to educate myself (and the private hospital birthing class was useless). So I just ended up with the birth that was convenient for my Obstetrician.”* (ID 223).

In the concept *‘I felt like I was part of a system’* women identified a lack of individualised care.

*“OB was there for appointments and care if I asked but I felt like I was part of a system. I’ve changed OB’s for this pregnancy, however they all seem much the same. Part of me wishes that I would just go public and go through the Midwife program. It was my Calmbirth course and the Midwife on shift that night that contributed to my good experience - not the OB (who’s stand in didn’t even turn up and I ended up with someone random delivering).”* (ID 252).

Finally, some women found the appointments to be limiting and short in duration in the concept *‘Each appointment and scan was very short’ “Very busy ob / appointments were very rushed”* (ID 322).

*“My main health care provider paid no attention to me as a person. He didn't remember my name until I was over seven months pregnant. Every visit was 2-3 minutes long and I was physically ushered out the door if I asked questions that took us over that time.”* (ID 1261).

### Privately practising midwife

Women who were cared for by Privately Practising Midwives (PPMs) highlighted receiving attentive care attuned to their individual birth preferences and feeling they were empowered as an active decision-maker in care options. This model of care received the greatest number of positive items of coding with 95.93% of all items of coding expressing benefits and the fewest number of limitations, with 4.07% of items of coding.

#### Strengths

There were three subcategories in the PPM strengths category, two discussed in previous models of care* ‘Having a great provider I know’ and ‘Woman-centered care’ and a subcategory unique to PPM, ‘Having the gold standard of maternity care’. The subcategory ‘Having the gold standard of maternity care’* had four concepts, with one concept, ‘This model should be the standard and accessible to all’ also present in MGP. The three concepts in this subcategory unique to PPM will now be discussed.

Many women expressed how positive they felt both the level of care and value of PMM was in the concept *‘I couldn’t rate my midwife more highly’*.

*“Extremely personable! Home visits were like having tea with a friend but very professional. Her knowledge and empathy made me feel safe and protected. She respected all of my decisions. She reminded me often that I didn’t need her help when it came to birthing my child, but she was there if I wanted it (or did need it).”* (ID 1504)

Although PPM requires out of pocket costs, women highlighted how valuable the care was in the concept *‘Best value for money ever spent’*.

*“Every woman should have access to this type of care. We were very fortunate to scrape together the money to pay for our midwife.”* (ID 1497).

In the concept ‘*Postpartum support’* women identified the benefit of the increased support following birth which occurs up to six weeks.

*“The level of post natal care was incredible. My midwife came every day post birth for about 10 days. I felt very well supported.”* (ID 223).

#### Limitations

There were two subcategories in the limitations category of PPM, *‘Bound by policies and protocols’ which has been presented in standard care and ‘I wasn’t able to form much of a meaningful relationship’* which was also present in MGP.

In the subcategory *‘I wasn’t able to form much of a meaningful relationship’* there were two concepts unique to PPM ‘*I had big hopes for my private midwife’* and *‘Impersonal care concerning birth preferences’.*

In the concept, *‘I had big hopes for my private midwife’*, women expressed they had high expectations for private midwifery care however felt disappointed in the level of care they received. One woman commented, *“I had big hopes for my private midwife but it did not go as well as I hoped it would.”* (ID 1538).

*“I had a private midwife for my second pregnancy which I thought would be great however I was constantly needing to remind her about different tests/things I needed, I felt she was distracted a lot during appointments.”* (ID 1537).

Some women noted feeling their midwife did not understand nor respect their involvement in their birthing wishes in the concept *‘Impersonal care concerning birth preferences’*. One woman commented *“I felt I had expressed what was important to me, but it was not followed”* (ID 1529)*,* while another women said, *“Didn’t ask enough questions on what I’d like my birth space to be like it would have been nice to be promoted about that from my main healthcare provider”* (ID 1492).

## Discussion

The findings of this study indicate that women across the models of care wished for continuity of carer. Women in fragmented models voiced disappointment when they were unable to, or prohibited from, accessing a model that provided continuity. For women in continuity models there was disappointment when there were disruptions in the provision of continuity or when there was a lack of connection in the relationship with the midwife or doctor.

The women in fragmented models highlighted a lack of continuity as a failing in the model of care they accessed, which contributed to the depersonalisation they also experienced. Midwifery continuity of care has been shown to improve perinatal outcomes, decrease interventions in labour, increase spontaneous vaginal birth rates, increase women’s satisfaction [4] and be cost effective for health services [26, 27]. Women who have experienced maternity care in Australia show a preference for midwifery models of care when considering their future maternity choices [20, 28]. National and State Australian maternity strategic directions identify the need for women to have access to midwifery continuity of care [6, 29]. However, barriers to upscaling midwifery continuity of care models remain and across Australia only 14% of models are midwifery continuity of care provided in a public hospital [1]. Upscaling midwifery CoC models requires a collaborative commitment to organisational change from Government, policy makers, managers, doctors, midwives and consumers to ensure sustainability and effectiveness [30] and further research is needed to identify the barriers and facilitators for upscaling midwifery models of care to ensure all women have access to continuity of care with a known midwife.

Women who had received care from privately practising midwives provided the highest percentage of comments on strengths of the model and lowest percentage of comments about limitations. Women described this model as the gold standard of maternity care and highlighted the involvement of partners and family in their care. This is supported by qualitative research from Western Australia where women stated a preference for the family centred, holistic and individualised care they received when choosing care through a privately practising midwife [31]. Studies from Queensland have found women receiving private midwifery care had higher spontaneous labour and vaginal births, used less pharmacological pain relief and had fewer caesareans compared to national population rates [32, 33]. There are ongoing challenges for PPM’s in Australia, such as being more likely to be reported to regulators [34] and a lack of professional indemnity insurance for homebirth [32]. There is limited Australian national data on the volume of PPM models of care [1] and the number of hospitals offering visiting access to PPMs across Australia [33]. There is great scope for upscaling PPM care across Australia.

Women in this study highlighted the counterintuitive aspect of not having access to continuity of care when their pregnancy has been identified as ‘high risk’. Women mentioned the frustration of seeing multiple providers and receiving conflicting medical advice, with some women feeling like they were receiving unsafe, inadequate care. This is supported by an integrative review on women with complex pregnancies which found women with higher risk often fell through the gaps of maternity services [5]. Often midwifery models of care are only available for women deemed low-risk, yet studies have shown the positive benefits of providing an all-risk midwifery continuity of care model [35]. A randomised control trial undertaken in the UK with women with risk factors for pre-term birth found no increased maternal or neonatal morbidity or mortality in the midwifery continuity of care group, however babies born in this model had significantly more skin to skin contact after birth and greater breastfeeding rates [36]. In Australia, a midwifery CoC model for First Nations women was found to decrease pre-term births and provide culturally safe care [37]. In this study women with risks identified during the pregnancy expressed an overwhelming need for relational care. While research suggests the benefits of midwifery continuity of care for women of all risk, further research on outcomes for women with complex pregnancies is needed to support the expansion of continuity of care programs.

Of significance is that women who received GP shared care commented on experiencing two sides in their maternity care and a strong desire for continuity across the continuum. Women who had known and trusted their GP prior to pregnancy, or who were able to form a personal relationship with their GP, noted overall positive birth experiences. This is supported in rural areas of Australia where women report high levels of satisfaction in GP care [38]. A scoping review undertaken in Australia that summarized women’s experiences and the clinical and neonatal outcomes of maternity services found significant gaps in existing research on GP shared care [39]. Despite GP shared care being the most frequently discussed model during the first antenatal visit [40], this model has not been directly compared against other available models. Further research is needed to explore the experiences and outcomes of women in GP shared care whilst being mindful that this study identified women’s difficulties in their GP not having sufficient knowledge about pregnancy and the division felt between community and hospital care.

The limitations of the continuity of care models were twofold; when the continuity became disrupted and when there was a lack of connection in the relationship with the provider. Women in this study found their continuity of care was disrupted when they were unable to receive care from their provider due to not being on call during their labour or no longer working in the model. This has been recognised by previous research as a challenging aspect for women experiencing continuity of care [41]. Further research is needed to explore how to navigate the work life balance of MGP midwives with the experiences of women.

As continuity of midwifery care is relational, some women in this study highlighted the disappointment when there was a lack of connection with their midwife. A study from New Zealand found midwives and women were unsure how to navigate the continuity of care relationship complexities that may result from a lack of trust or connection [42]. Midwives reported hoping the relationship would improve over time or having difficult conversations to find resolution [42]. Further research is needed to explore pathways for women to be able to express their concerns during their care and find resolution or to choose a different provider.

In this study women across all the models of care identified the benefit of having a student midwife allocated to them, who could also be present during labour and birth when their CoC midwife or doctor was unavailable. This is supported by Tickle et al. [43] who found women rated their experience with a student midwife as ‘better than they hoped for’, especially if they were present for their labour and birth. Student midwives also benefited and enjoyed following women through their midwifery education and many wish to work in continuity of care models when graduated [44, 45].

### Strengths and weaknesses of the study

A strength to this study is the large number of responses received, comparable to previously published national birth experience surveys in other high resource countries [46–49]. However, there were limited responses from women who read and wrote in languages other than English, despite translating surveys into seven languages and using targeted social media advertising. Fewer First Nations women were represented in the cohort compared to Australian maternity statistics. A strength was the representation of women from all States and Territories of Australia that was representative of national Australian data [20, 22].

The survey was only available online which was a limiting factor for women without internet access or devices needed to access the internet.

As respondents chose to respond to the survey and the question explored in this study, the findings are not generalisable to the wider population. More comments were received from women who received midwifery continuity of care compared to the other models of care.

## Conclusions

This study has explored women’s experiences of maternity models of care and used a strengths and limitations framework to group the data. The higher the level of continuity of care across the entire maternity care experience, including postnatal care, the more positive women were about the model. The women in this study wished for continuity of carer and were disappointed when they were unable to access a model that provided CoC. Women were disappointed with CoC when there were disruptions in continuity or the ability to make a connection with their provider. The strengths of continuity of care, specifically private midwifery, should be recognised and the limitations for women in high risk maternity care investigated and prioritised by policy makers and managers in health services. Designing a maternity service that is women-centred and therefore inclusive of women’s experiences should be a priority for policy maker and managers in health services.

## Data Availability

The data that support the findings of this study are available on request from the corresponding author HK. The data are not publicly available due to them containing information that could compromise research participant privacy/ consent.

## Abbreviations

BESt: Australian birth experience study
CoC: Continuity of care
GPS: GP Shared care
HCP: Health care provider
HRC: High risk care
MGP: Midwifery group practice
POB: Private obstetric
PPM: Privately practising midwifery
SC: Standard public hospital care
